# In vitro transcription of self-assembling DNA nanoparticles

**DOI:** 10.1038/s41598-023-39777-0

**Published:** 2023-08-10

**Authors:** Chang Yong Oh, Eric R. Henderson

**Affiliations:** 1 Department of Biochemistry and Molecular Biology, Iowa State University, Ames, IA 50011 USA; 2 Department of Genetics, Development, and Cell Biology, Iowa State University, Ames, IA 50011 USA

**Keywords:** Biological techniques, Biotechnology, Genetics, Structural biology

## Abstract

Nucleic acid nanoparticles are playing an increasingly important role in biomolecular diagnostics and therapeutics as well as a variety of other areas. The unique attributes of self-assembling DNA nanoparticles provide a potentially valuable addition or alternative to the lipid-based nanoparticles that are currently used to ferry nucleic acids in living systems. To explore this possibility, we have assessed the ability of self-assembling DNA nanoparticles to be constructed from complete gene cassettes that are capable of gene expression in vitro. In the current report, we describe the somewhat counter-intuitive result that despite extensive crossovers (the stereochemical analogs of Holliday junctions) and variations in architecture, these DNA nanoparticles are amenable to gene expression as evidenced by T7 RNA polymerase-driven transcription of a reporter gene in vitro. These findings, coupled with the vastly malleable architecture and chemistry of self-assembling DNA nanoparticles, warrant further investigation of their utility in biomedical genetics.

## Introduction

Self-assembling nucleic acid nanosystems (i.e., DNA nanoparticles) provide an architecturally and chemically malleable platform for a wide range of applications^1–8^. Exemplary uses of DNA nanoparticles include targeted drug delivery^1–3,5,6,8–16^, biosensing^1,3,5,7,17^, nanoelectronics^3,6,12,18^, bioimaging^1^, nanorobotics^2,3,5,12^, nanophotonics^5,7,17,18^, CpG triggered immunostimulation^2,5,11,12^, synthetic nanopore formation^1^, templates for CRISPR-mediated in vivo genomic integration followed by gene expression^19^ and, recently, as gene expression vehicles in cell culture^20^. Although DNA is inherently immunogenic, it can be chemically modified to tune its biocompatibility^1–3,7,8^. Thus, there may be additional opportunities in biomedicine for functional DNA nanoparticles with nuanced immunological properties.

The M13 bacteriophage ssDNA genome has been the workhorse scaffold for DNA nanoparticles constructed by the method of DNA origami^21^. As methods and applications of DNA nanotechnology have expanded there has been a corresponding increased interest in the use of scaffolds other than M13 ssDNA^22,23^. Nonetheless, even with the availability of a larger library of single-stranded scaffolds now accessible, the vast majority of DNA nanoparticle applications focus on the use of the scaffold as an engineering component for sculpting DNA nanoparticle architecture and function with less attention paid to its genetic code content^1,2,9,13^.

In this report, we return focus to the genetic information in the scaffold of DNA nanoparticles to evaluate whether this information may still be biologically active even when folded and containing numerous crossover junctions. We find that variously sculpted DNA nanoparticles containing a full gene cassette can be transcribed in vitro. This finding suggests that it may be feasible to employ self-assembling DNA nanosystems as a platform for delivery of biologically active, intentionally sculpted, and camouflaged gene cassettes to specific locations in cell culture and even in vivo wherein they may be expressed with or without the requirement of chromosomal insertion^19,20^.

## Materials and methods

Gene-bearing DNA origami scaffolds were prepared as previously described^24^, summarized as follows.

### Standard PCR

Primers for amplification of the Green Fluorescent Protein (GFP) gene were designed using SnapGene and purchased from Integrated DNA Technologies (IDT, Coralville, IA, USA). The sequence of each primer is listed in Supplementary Table 1. Phusion® polymerase for the PCR reaction was purchased from New England Biolabs (NEB, Ipswich, MA, USA).

For the generation of a duplex gene containing all components for transcription, each PCR reaction mixture was prepared in 50 µL final volume, composed of 1 × Phusion® HF buffer (NEB), 200 nM dNTP mix (NEB), 500 nM sense primer (T7EGFP sense = undesired sense strand), 500 nM antisense primer (T7EGFP anti = desired antisense strand), 10 ng plasmid template (pCMV-T7-EGFP; Addgene, Watertown, MA, USA), 0.5 µL Phusion® DNA polymerase, and nuclease-free water to volume. Each PCR was performed using the following thermocycler steps: 30 s at 98 °C, 30 s at 58 °C, and 1 min at 72 °C for 30 cycles^24^.

For the generation of duplex gene missing promoters, each PCR reaction mixture was prepared in 50 µL final volume, composed of 1 × Phusion® HF buffer (NEB), 200 nM dNTP mix (NEB), 500 nM sense primer (RT-sense), 500 nM antisense primer (T7EGFP anti), 10 ng pCMV-T7-EGFP (Addgene), 0.5 µL Phusion® DNA polymerase, and nuclease-free water to volume. Each PCR was performed using the following thermocycler steps: 30 s at 98 °C, 30 s at 59 °C, and 1 min at 72 °C for 30 cycles^24^.

The reaction products were mixed with 6 × loading dye (15% Ficoll®-400, 60 mM EDTA, 19.8 mM Tris–HCl, 0.48% SDS, 0.12% Dye 1, 0.006% Dye 2, pH 8 at 25 °C; NEB) and then loaded onto a 1% agarose gel pre-stained with SYBR safe DNA dye (Invitrogen, Waltham, MA, USA). Electrophoresis was carried out at 8 V/cm for 1 h. The SYBR Safe-containing DNA was visualized using a 490 nm wavelength (blue) transilluminator and an amber filter^24^.

### Asymmetric PCR (aPCR)

Primers used in aPCR were identical to those used in standard PCR. Along with sense and antisense primers, a 3’ terminal modified primer (3’ T7EGFP blocker) was used. The 3’ T7EGFP blocker was designed using SnapGene and purchased from IDT. Its sequence is listed in Supplementary Table 1.

For the generation of a scaffold containing all components for transcription, each aPCR reaction was carried out in 50 µL total volume, composed of 1 × LongAmp® Taq buffer (60 mM Tris-SO_4_, 20 mM (NH_4_)_2_SO_4_, 2 mM MgSO_4_, 3% glycerol, 0.06% IGEPAL® CA-630, 0.05% Tween® 20, pH 9.1 at 25 °C) from NEB, 500 nM T7EGFP anti, 25 nM T7EGFP sense, 475 nM 3’ T7EGFP blocker, 300 nM dNTP mix from NEB, 10 ng double-stranded GFP gene (dsT7EGFP; generated by standard PCR), 2 µL LongAmp® Taq DNA polymerase (NEB), and nuclease-free water to final volume. Each aPCR was performed using the following thermocycler steps: 30 s at 94 °C, 30 s at 58 °C, and 2 min at 65 °C for 25 cycles^24^.

For the generation of a scaffold containing all components for transcription except the promoter element, each PCR reaction was carried out in 50 µL total volume, composed of 1 × LongAmp® Taq buffer (60 mM Tris-SO_4_, 20 mM (NH_4_)_2_SO_4_, 2 mM MgSO_4_, 3% glycerol, 0.06% IGEPAL® CA-630, 0.05% Tween® 20, pH 9.1 at 25 °C) from NEB, 1 µM T7EGFP anti, 20 nM RT sense, 300 nM dNTP mix from NEB, 10 ng double-stranded GFP gene devoid of promoters (dsT7EGFP -T7; generated by standard PCR), 2 µL LongAmp® Taq DNA polymerase (NEB), and nuclease-free water to final volume Each aPCR was performed using the following thermocycler steps: 30 s at 94 °C, 30 s at 59 °C, and 2 min at 65 °C for 25 cycles^24^.

The reaction product was loaded onto a 1% agarose gel pre-stained with 1 × SYBR Safe (Invitrogen), electrophoresed, and visualized as above.

### Double-stranded DNA (dsDNA) purification

A Zymoclean Gel DNA Recovery Kit (Zymo Research, Irvine, CA, USA) was used to extract dsDNA from agarose gels. Gel bands containing target dsDNA were removed using a clean razor blade. Three times the gel slice volume of the provided agarose dissolving/binding buffer was added to each gel fragment and incubated at 55 °C on a heat block for 15 min. Each dissolved gel solution was transferred to a provided silica-based spin column and centrifuged at 10,000 relative centrifugal force (rcf) for 60 s in a table-top centrifuge. 200 µL of ethanol-based DNA wash buffer was added to each spin column and centrifuged at 10,000 rcf for 30 s. A washing step was repeated before centrifuging at 10,000 rcf for 60 s for the complete removal of ethanol. Flow-through from all steps was discarded. After transferring each spin column to a clean microcentrifuge tube, 6–20 µL of the provided elution buffer (10 mM Tris–HCl, 0.1 mM EDTA, pH 8.5) was added directly to the matrix of each spin column followed by centrifugation at 10,000 rcf for 60 s for DNA collection. A fraction of each purified dsDNA was mixed with 6 × loading dye (NEB) and loaded onto 1% agarose gel pre-stained with 1 × SYBR safe (Invitrogen). The gel was run at 8 V/cm for 1 h. The yield of the purified dsDNA sample was evaluated by measuring band intensities relative to a known control using GelAnalyzer 19.1 available at www.gelanalyzer.com (accessed on 19 August 2021)^24^.

### Single-stranded (ssDNA) purification

A Zymoclean Gel RNA Recovery Kit from Zymo Research was used to purify ssDNA from agarose gels. The gel bands containing target ssDNA were excised with a clean razor blade. Three times the gel slice volume of the provided agarose dissolving/binding buffer was added to each excised gel band and melted at 55 °C on a heat block for 15 min. Each dissolved gel solution was transferred to a provided silica-based spin column and centrifuged at 12,000 rcf for 2 min. 400 µL RNA Prep buffer was added to each spin column followed by centrifugation at 12,000 rcf for 1 min. Washing was carried out by the addition of 800 µL ethanol-based wash buffer followed by centrifugation at 12,000 rcf for 30 s. After repeating the washing step with 400 µL ethanol-based wash buffer, each spin column was centrifuged at 12,000 rcf for 2 min to remove residual ethanol. Flow-through in all steps was discarded. After transferring each spin column to clean microcentrifuge tubes, 6–20 µL of provided nuclease-free water was added directly to the column matrix, and the spin columns were centrifuged at 10,000 rcf for 1 min for retentate collection. A fraction of each purified ssDNA was mixed with 6 × loading dye (NEB) and the yield was estimated by gel electrophoresis as described above^24^.

### DNA nanoparticle construction

DNA nanoparticles were designed using caDNAno (www.cadnano.org) and staples were purchased from IDT (Supplementary Tables 2 and 3). DNA nanoparticles were prepared by mixing single-stranded GFP gene (ssT7EGFP or ssT7EGFP -T7; generated by aPCR) to a final concentration of 91.4 nM and each staple to a final concentration of 457 nM in 1 × TAE buffer supplemented with 12.5 mM Mg(OAc)_2_ (TAEM) in a final volume of 50 µL. The staple set for each DNA nanoparticle is listed in Supplementary Table 4. The mixture was incubated at 90 °C for 10 min in a water bath followed by gradual cooling to room temperature. Products of this reaction were mixed with 6 × loading dye (NEB) and then loaded onto 1% agarose gel containing 12.5 mM Mg(OAc)_2_ pre-stained with 1 × SYBR Safe DNA dye (Invitrogen). Electrophoresis was carried out in TAEM buffer at 6 V/cm for 90 min. The gel was visualized as above. DNA origami was purified using a Freeze ‘N Squeeze™ DNA Gel Extraction Spin Column (Bio-Rad, Hercules, CA, USA). Gel bands containing target DNA origami were sliced and removed using a clean razor blade, then transferred to Freeze ‘N Squeeze™ DNA Gel Extraction Spin columns. Spin columns containing target DNA origami gel slices were incubated at − 20 °C for 5 min followed by centrifugation at 13,000 rcf in a table-top centrifuge for 3 min at room temperature. The concentration of the purified DNA origami samples was measured using a NanoDrop™ instrument.

### In vitro transcription (IVT)

All IVT reactions were carried out in 20 µL total volume using a HiScribe® T7 Quick High Yield RNA Synthesis Kit (NEB). Each reaction contained 10 µL NTP buffer mix (10 mM each NTP; NEB), 10 ng DNA template (linearized GFP plasmid (LpCMV-T7-EGFP), dsT7EGFP, ssT7EGFP, each DNA nanoparticle (designated as described in Table 1 below and in the legend for Fig. 2b), 2 µL T7 RNA polymerase mix, and nuclease-free water to volume. Each reaction was carried out at 37 °C for 2 h. For T7GHL FS, T7GHL PO, and T7GHL HS, a reaction time course of 30, 60, 90, and 120 min was carried out for a preliminary comparison of the relative transcription rates of these constructs and substrate longevity.

**Table 1:** Names of DNA scaffolds and DNA nanoparticles used in this study.

Name	Contents
ssT7EGFP	Single-stranded antisense strand for nanoparticle scaffold
ssT7EGFP -T7	Single-stranded antisense strand for nanoparticle scaffold lacking the T7 promoter
T7GHL PO	DNA nanoparticle with a linear duplex promotor and single-stranded GFP gene; variant of T7-based GFP gene Honeycomb structure with a Linear promoter, the Promoter Only being in duplex form within the structure
T7GHL HS	DNA nanoparticle with a linear duplex promotor and a half complement of staples (i.e., loosely folded); variant of T7-based GFP gene Honeycomb structure with a Linear promoter, the structure folded with a Half-Set of staples
T7GHL FS	DNA nanoparticle with a linear duplex promotor and a full complement of staples (i.e., tightly folded); variant of T7-based GFP gene Honeycomb structure with a Linear promoter, the structure folded with a Full-Set of staples
T7GHL BP	DNA nanoparticle with a linear duplex promotor that is buried within a folded architecture with a full complement of staples (i.e., buried promoter, tightly folded); variant of T7-based GFP gene Honeycomb structure with a Linear promoter; the structure with Buried Promoter inside the structure
T7GHL -T7	DNA nanoparticle with a full complement of staples but lacking the T7 promoter sequence; variant of T7-based GFP gene Honeycomb structure with a Linear promoter, the structure lacking (-) T7 promoter

### Purification of IVT products

Following IVT, 30 µL of nuclease-free water was added to each IVT reaction product to increase the reaction volume. Each IVT product was then purified using a Monarch® RNA Cleanup Kit (NEB). 100 µL of RNA binding buffer was added to each 50 µL IVT product followed by the addition of 150 µL of absolute ethanol. Each mixture was then transferred to a provided silica-based spin column and centrifuged in a table-top centrifuge. 500 µL of ethanol-based DNA wash buffer was added to each spin column and centrifuged as above. The washing step was repeated once more and the flow-through from all steps was discarded. Each spin column was transferred to a clean microcentrifuge tube and 10 µL of the provided nuclease-free water was added directly to the matrix of each spin column followed by centrifugation for DNA collection. All centrifugation steps were carried out in 16,000 rcf for 60 s.

### Evaluation of IVT products

IVT products from all DNA samples were analyzed by gel electrophoresis. Purified GFP PCR and linearized GFP plasmid IVT products were diluted 100-fold to avoid over-staining. Purified RNA products were mixed with an equal volume of 2 × RNA loading dye (95% formamide, 0.02% SDS, 0.02% bromophenol blue, 0.01% Xylene Cyanol, 1 mM EDTA; NEB). Samples were heated to 70 °C for 10 min prior to gel loading. Electrophoresis was carried out at 8 V/cm for 1 h. The gels were post-stained with 1 × TAE solution containing 1 × SYBR gold (Invitrogen) for 2 h. The stained gels were visualized using a 490 nm wavelength transilluminator and an amber filter.

### Reverse transcription PCR (RT-PCR)

RNA templates for RT-PCR were prepared by carrying out an IVT reaction using each DNA template (LpCMV-T7-EGFP, dsT7EGFP, ssT7EGFP, T7GHL PO, T7GHL HS, T7GHL FS, and T7GHL BP) as described above. To minimize the possible background signal caused by the presence of residual DNA template, 1 ng of each DNA template (rather than 10 ng) was used for these reactions, and the incubation time was extended to overnight to maximize RNA production under these conditions. Following IVT, DNA was hydrolyzed by treatment with DNase. A 50 µL DNase mixture was prepared by mixing 20 µL IVT product with 2 µL RNase-free DNase-I and nuclease-free water to volume. All DNase reactions were carried out at 37 °C for 30 min. DNase-treated RNA products were purified using a Monarch® RNA Cleanup Kit (NEB) as described above. Primers for amplification of the GFP gene were designed using SnapGene and purchased from IDT. The sequence of each primer is listed in Supplementary Table 1. RT-PCR was performed using a OneTaq® One-step RT-PCR kit (NEB). Each RT-PCR mixture was carried out in 50 µL final volume, containing 1 × Quick-Load® OneTaq One-step reaction mix (1.6 mM MgCl_2_, 250 nM dNTP mixture), 400 nM sense primer (RT-sense), 400 nM antisense primer (RT-anti), 1 µL of each purified RNA product, 1 × OneTaq® One-step enzyme mix (ProtoScript® II reverse transcriptase, OneTaq® Hot Start DNA polymerase, Murine RNase inhibitor, and stabilizer), and nuclease-free water to volume. To assess any contribution to PCR signal from residual DNA resulting from incomplete DNase hydrolysis, negative controls were prepared in parallel. These reactions contained the same components as RT-PCR mixtures but substituted OneTaq® Hot Start DNA polymerase for the OneTaq® One-step enzyme mix (i.e., lacking reverse transcriptase altogether). In this case, any signal represented the amplification of residual DNA remaining after DNase treatment. Reactions were carried out by treating each RT-PCR mixture at 48 °C for 30 min followed by PCR. Each PCR was performed using the following thermocycling steps: 30 s at 94 °C, 30 s at 60 °C, and 1 min at 68 °C for 15 cycles. Each product was loaded onto a 1% agarose gel pre-stained with SYBR-safe DNA dye (Invitrogen). The gel was electrophoresed and visualized as above.

### Transmission electron microscopy (TEM)

TEM was performed as previously described^25^. Briefly, samples for TEM imaging were prepared in a concentration range of 0.5 nM to 5 nM. 12 μL of the sample was placed on glow-discharged carbon-coated 400 mesh copper TEM grids. After two minutes of incubation, the sample solution was removed using filter paper and replaced with 12 μL of freshly prepared uranyl formate negative staining solution. The stain was removed after 30 s, and the grids were air-dried. TEM images were acquired at 25,000 × magnification using a JEOL 1230 TEM (Peabody, Ma, USA) equipped with a Gatan Inc. 2 k × 2 k Ultrascan camera (Pleasanton, CA, USA)^25^.

## Results

### DNA nanoparticle construction

**Figure 1:**
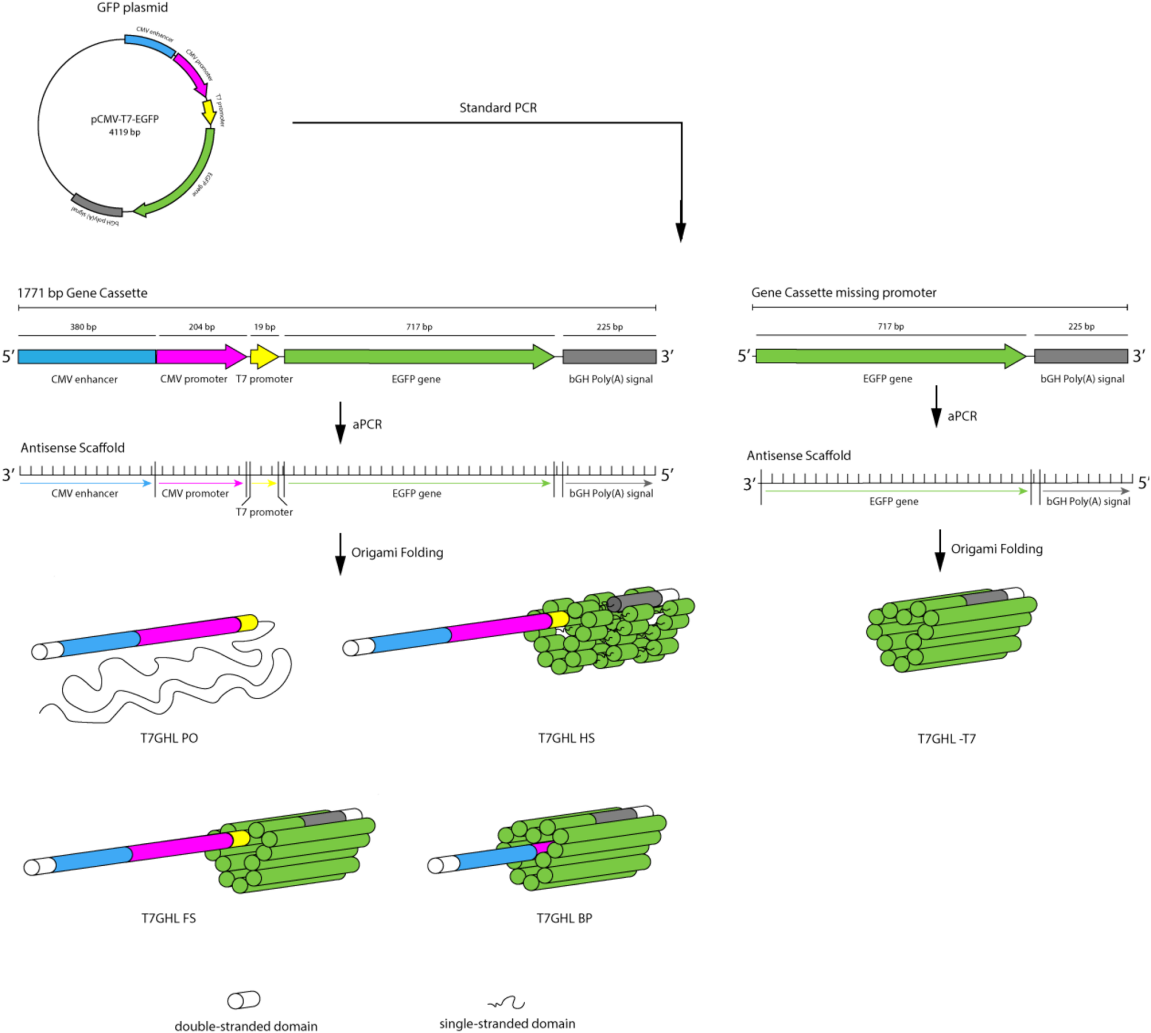
Illustration of the gene construct used in this study and the process of scaffold and DNA nanoparticle generation via aPCR and DNA origami, respectively, along with schematic diagram of different DNA nanoparticles used in the study. Primary variations include the number/position of crossovers in the origami architecture and relative accessibility of the T7 RNA polymerase promoter region; the T7 promoter being located either on a linear duplex extending from the body of the nanoparticle, embedded within the nanoparticle, or absent altogether.

**Figure 2:**
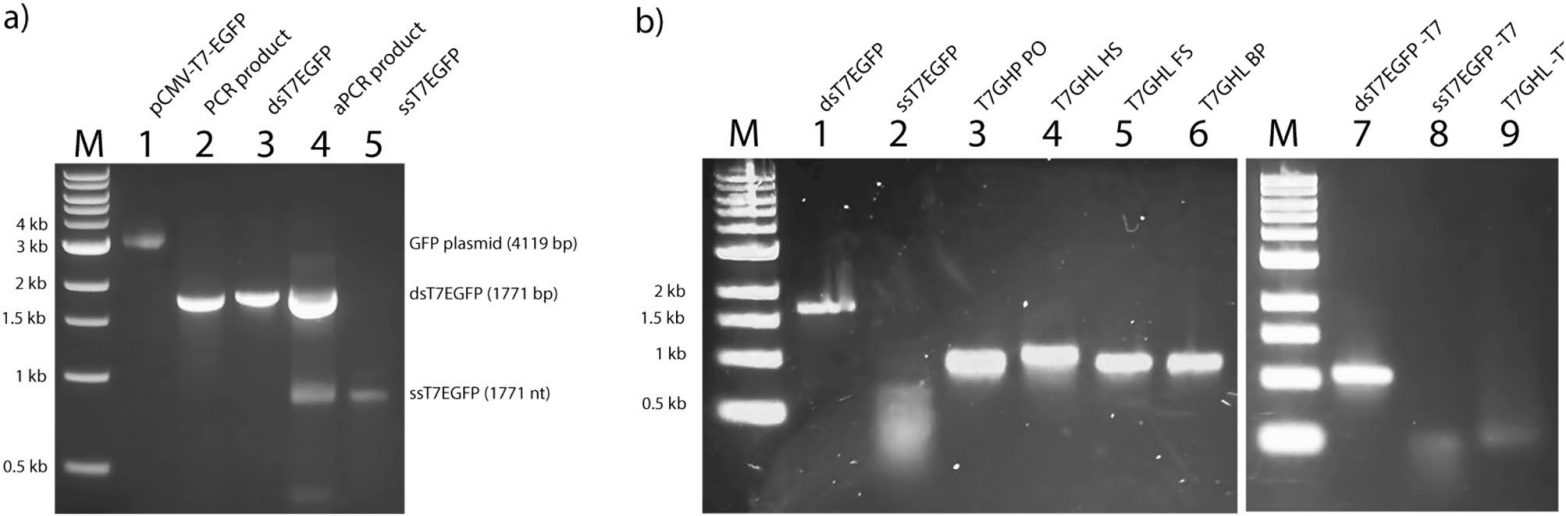
Agarose gel electrophoretic (AGE) result of DNA nanoparticle production. (**a**) ssDNA production. Representative non-denaturing agarose gel electrophoretic results of PCR and aPCR. M: molecular marker; 1: GFP plasmid template for PCR (pCMV-T7-EGFP); 2: unpurified PCR product; 3: purified PCR product (dsT7EGFP); 4: unpurified aPCR product; 5: purified aPCR product (ssT7EGFP). The bands at approximately 3 kb, 1.7 kb, and 0.9 kb represent GFP plasmid, double-stranded GFP gene, and single-stranded GFP gene, respectively. **(b)** Generation of DNA nanoparticles from GFP gene-containing scaffolds ± the T7 promoter; ssT7EGFP and ssT7EGFP -T7. Representative agarose gel electrophoresis display of the DNA nanoparticles used in this study. M: molecular marker; 1: dsT7EGFP; 2: ssT7EGFP; 3: T7GHL PO (DNA nanoparticle with a linear duplex promoter and single-stranded gene); 4: T7GHL HS (DNA nanoparticle with a linear duplex promoter and partially folded gene with half a set of staples); 5: T7GHL FS (DNA nanoparticle with a linear duplex promoter and a full set of staples); 6: T7GHL BP (DNA nanoparticle with a linear duplex promoter buried inside the fully folded gene); 7: dsT7EGFP -T7 (double-stranded GFP gene without T7 promoter); 8: ssT7EGFP -T7 (single-stranded GFP gene without T7 promoter); 9: T7GHL -T7 (fully folded DNA nanoparticle without T7 promoter). For lanes 1 and 2, the band at approximately 1.7 kb represents dsT7EGFP, and the smear that runs from approximately 0.7 kb to 0.3 kb represents ssT7EGFP. The bands around 1 kb for lanes 3, 4, 5, and 6 represent successfully self-assembled DNA nanoparticles. For lanes 7 and 8, the band at approximately 1.1 kb represents dsT7EGFP -T7 and the smear that runs from 0.5 kb to 0.4 kb represents ssT7EGFP -T7. The band at approximately 0.6 kb in Lane 9 represents the successfully self-assembled DNA nanoparticle lacking the T7 promoter.

The overall process of generating the DNA nanoparticles used in this study is illustrated in Fig. 1. For the construction of potentially transcription-capable DNA nanoparticles, a custom scaffold was synthesized that contained the required gene sequence elements for expression. These scaffolds included a T7 promoter, the GFP gene, and a poly A signal (a CMV promoter and enhancer, not relevant to the present study, were also present). Conventional PCR followed by asymmetric PCR (aPCR) was performed to produce the single-stranded scaffold containing the GFP gene cassette, designated ssT7EGFP and ssT7EGFP -T7, the latter lacking the T7 promotor as a negative control (Table 1). The same primers were used for both PCR and aPCR, but in the case of the aPCR, an unequal ratio of primers was employed to enhance the production of the desired strand (the genetically defined antisense strand). A 3’ terminal blocker was used along with both sense and antisense primers to optimize the production of the desired strand by aPCR. Electrophoretic analysis of the products of this process revealed two bands estimated to be ~ 1700 bp and ~ 900 bp in length as determined by co-migration with dsDNA markers (Fig. 2a). Both the dsT7EGFP and the ssT7EGFP are 1771 nucleotides in length. However, the single-stranded form migrates faster under non-denaturing conditions presumably due to internal base-pairing. Thus, we interpret the strong DNA band at ~ 1700 bp as being dsT7EGFP and the band at approximately 900 bp to be the desired ssT7EGFP. This interpretation was corroborated by the difference in SYBR Safe DNA dye color and staining efficiency^24^ and further validated by the use of the presumed ssDNA band for the successful construction of DNA origami nanoparticles (below).

The program caDNAno^26^ was employed to design the DNA nanoparticles used in this study. ssT7EGFP was used to construct nanoparticle architectures designated T7GHL PO, T7GHL HS, T7GHL FS, and T7GHL BP, and ssT7EGFP -T7 was used to construct T7GHL -T7 (Fig. 2b, and Supplementary Fig. 1). Details regarding the acronyms of the DNA nanoparticles are listed in Table 1. A schematic diagram of the structures of each DNA nanoparticle is also provided in Fig. 1. These nanoparticles were designed to form a cylindrical shape, maintaining a crossover pattern that was relatively consistent throughout various forms of this general structure. With the exception of a T7GHL BP in which the T7 promoter is buried within the nanoparticle architecture, the promoter of the GFP gene constructs was configured as a linear (i.e., no crossovers) double-stranded DNA helix adjacent to the cylindrical core (Supplementary Fig. 1). The intent of external positioning of the T7 promoter was due to a naïve assumption that it was advantageous to optimize accessibility to the promoter by T7 polymerase. To test the validity of this assumption the T7GHL BP construct was designed with the promotor sequestered inside the cylindrical architecture with crossover density consistent with the rest of the nanoparticle architecture. Single-stranded hairpins were positioned at the ends of each double helix in all constructs to facilitate the folding process (Supplementary Fig. 1).

**Figure 3:**
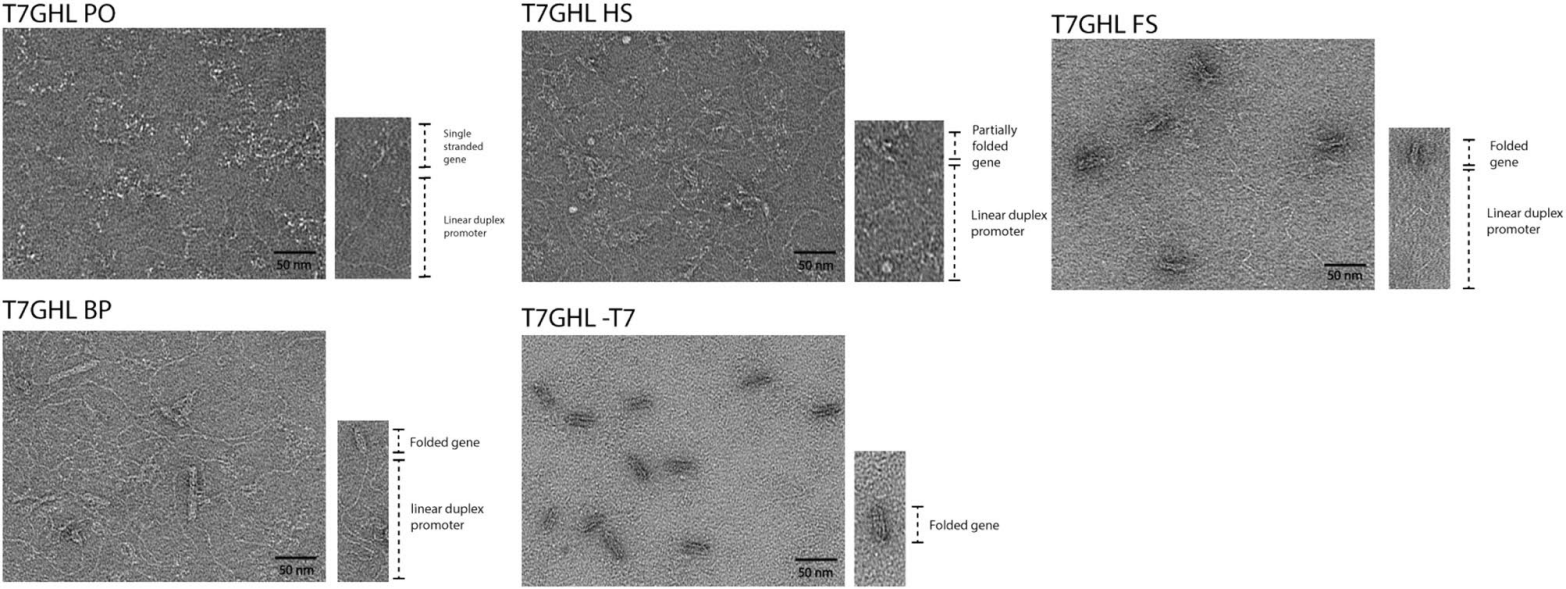
Verification of the structural integrity of gel-purified DNA nanoparticles by transmission electron microscopy (TEM). A representative DNA nanoparticle indicating key structural elements to the lower right of each representative TEM field. Single-stranded and loosely crosslinked domains appear as relatively amorphous consolidated networks whereas linear duplexes containing the T7 promoter appear as curved tails due to the greater persistence length of double-stranded DNA relative to single-stranded DNA^29^. Compact, highly crosslinked origami regions display the canonical multi-helix particle morphology.

To assess the efficiency of transcription as a function of the number of crossovers employed (“compactness”), DNA nanoparticles were constructed with varying staple density (Supplementary Fig. 1). Also, to evaluate whether the IVT products were the result of promoter-based transcription rather than random priming, one of the nanoparticles was constructed with a scaffold that was devoid of any T7 promoter sequence elements. Electrophoretic analysis of the DNA nanoparticle constructs revealed band shifts that were distinct from ssT7EGFP and dsT7GFP (Fig. 2b), indicating the successful self-assembly of the intended DNA nanoparticles. To minimize contamination from staples and incomplete or aberrantly folded structures that might hamper the interpretation of IVT data^27^, all constructs were gel purified (Fig. 2b). Freeze-and-squeeze gel purification is a commonly used method to obtain intact ultrapure DNA nanoparticles^19,28^. Thus, the freeze-and-squeeze gel purification method was employed for the purification of DNA nanoparticles used in the study. Confirmation of the retention of the structural integrity of the DNA nanoparticles used in this study was obtained by TEM imaging of gel-purified samples (Fig. 3).

### In vitro transcription (IVT)

IVT analysis was performed using the T7 promotor and a commercial T7 polymerase IVT kit (NEB). Positive controls included a linearized GFP plasmid (LpCMV-T7-EGFP) and the linear duplex GFP PCR product (dsT7EGFP) to assess the expected maximum degree of RNA production. A single-stranded antisense strand (i.e., the bare origami scaffold; ssT7EGFP) was used as a negative control based on the reported requirement for a double-stranded T7 promoter for successful transcription^30,31^. The IVT reactions from positive controls (dsT7EGFP and LpCMV-T7-EGFP) generated substantial quantities of RNA products and, therefore, these products were diluted 100-fold for clear visualization by electrophoresis. Banding patterns on non-denaturing (native) and denaturing gels were virtually identical, thus, the bulk of these analyses was carried out under non-denaturing conditions. Size estimates were based on a calibrated RNA ladder from a commercial supplier (NEB).

**Figure 4:**
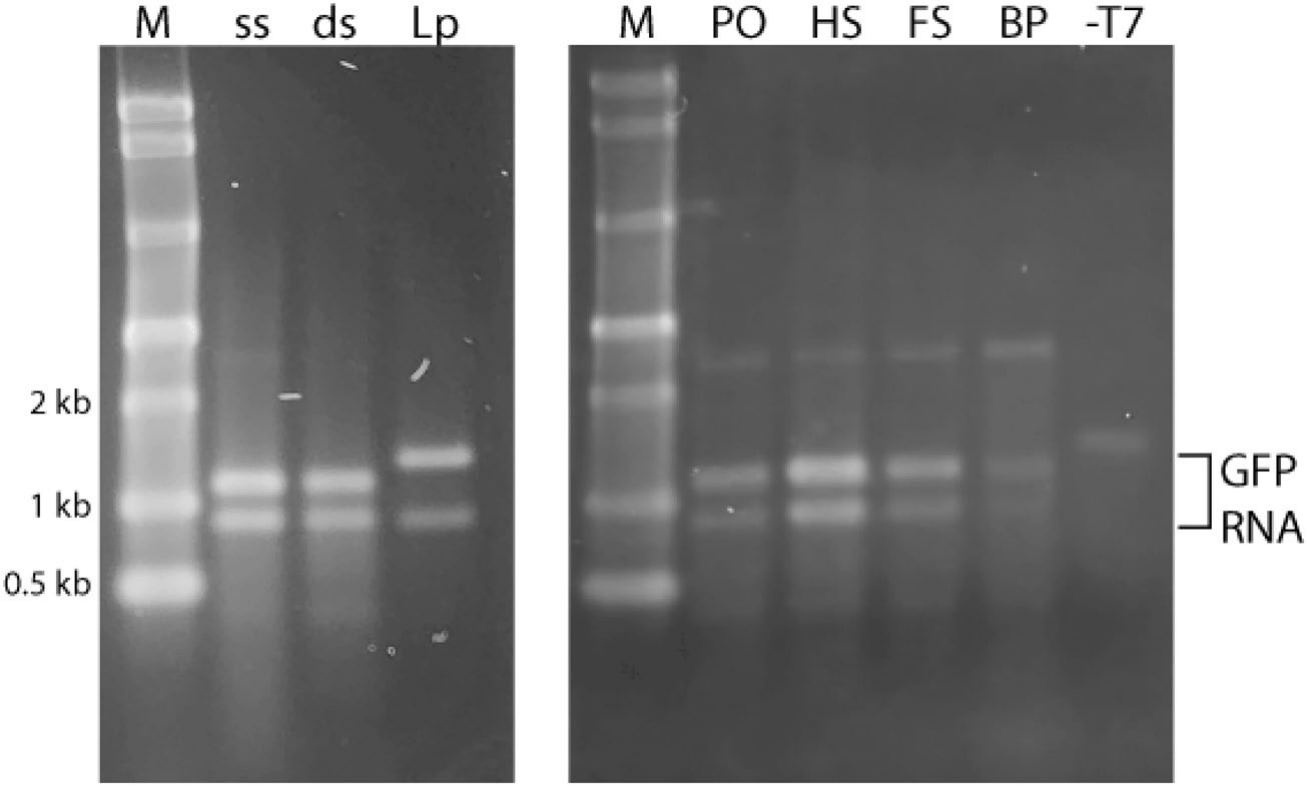
In vitro transcription of DNA nanoparticles. Representative non-denaturing agarose gel electrophoresis results of in vitro transcription reactions. M: molecular marker; ss: ssT7EGFP; ds: dsT7GFP; Lp: LpCMV-T7-EGFP (linearized GFP plasmid); PO: T7GHL PO; HS: T7GHL HS; FS: T7GHL FS; BP: T7GHL BP; -T7: T7GHL-T7. LpCMV-T7-EGFP and dsT7EGFP were positive controls. ssT7EGFP, T7GHL BP, and T7GHL -T7 were putative negative controls. The bands at approximately 0.9 kb, 1.1 kb, and 1.3 kb represent GFP RNA. The 0.9 kb band represents transcripts that terminate at a T7 RNA polymerase termination/stall sequence, known to allow a significant amount of read-through^32,33^. The 1.1 kb and 1.3 kb RNA products represent read-through transcripts, the latter’s greater length resulting from the linearized plasmid being 200 bp longer than the PCR-generated linear duplex. The bands of approximately 2.5 kb for most of the samples and 1.5 kb in the T7GHL -T7 lane result from the presence of residual DNA templates.

Electrophoretic analysis of IVT products revealed two RNA bands with approximate sizes of 1100 nucleotides (nt) and 900 nt (Fig. 4). IVT of linearized GFP plasmid resulted in RNA products with approximate sizes of 1300 nt and 900 nt. The larger product is consistent with the expected full-length transcript for linearized GFP plasmid which is 200 bp longer than the PCR-generated linear duplex, dsT7EGFP. Denaturing PAGE ruled out the possibility that the 900 nt product was a stable folded (i.e., internally base-paired) form of the 1100 nt long product. Previous studies have shown that sequence 5’ ATCTGTT 3’ can be responsible for the pausing or termination of T7 RNA polymerase^32,33^. The target DNA in these studies contains this sequence near the 3’ end and termination at this position would create an RNA product of the observed size. Thus, it is likely that the aforementioned termination sequence is responsible for the smaller of the two different RNA products observed. The electrophoretic results also revealed bands at approximately 2.5 kb in general and 1.5 kb in the T7GHL-T7 lane. Given that the templates for the IVT reaction did not exceed 2.5 kb (i.e., the 2500 nt product cannot be an RNA product because the templates were much shorter than 2 kb) and the ssRNA ladder used in Fig. 4 was not a dependable standard for measuring the size of dsDNA, it is reasonable to conclude that the bands around 2.5 kb represent residual DNA templates (Fig. 4).

Based on previous reports involving the study of the requirements of a duplex promoter for transcriptional activity, we assumed that a fully single-stranded promoter would preclude transcription initiation^30,31^. However, ssT7EGFP, the fully single-stranded antisense scaffold, was transcribed and produced RNA products that were indistinguishable from those produced by the positive controls. Sequence analysis revealed that portions of ssT7EGFP contain partial sequence complementarity to the promoter region. Previous studies have shown that partial complementarity of this nature is sufficient to support T7 promoter function^34^. Thus, our current hypothesis is that a foldback structure mediated by partial T7 promoter complementarity permitted low-level transcription initiation with purely single-stranded template DNA.

Electrophoretic analysis of IVT products from the structured DNA nanoparticles used in this study revealed banding patterns identical to the positive controls. All of the DNA nanoparticles tested produced identically sized RNA products except for the negative control DNA nanoparticle that was completely devoid of the T7 promoter element (Fig. 4). To assess whether DNA nanoparticles retain their structural integrity under IVT reaction conditions, T7GHL FS was incubated under IVT reaction conditions in the absence of T7 RNA polymerase and in the presence of increasing magnesium ion concentration (Mg^2+^; known to enhance the formation and stability of DNA origami nanoparticles^21^). The result was analyzed by gel electrophoresis. The banding pattern revealed no obvious change in DNA nanoparticle migration upon increasing Mg^2+^ concentration suggesting that the DNA nanoparticles used in this study maintain their structural integrity under the conditions used for IVT reactions (Supplementary Fig. 2). Moreover, we observed an inverse correlation between “compactness” (i.e., crossover density) of the DNA nanoparticles used in this study and RNA production. This strengthens the suggestion that the DNA nanoparticles remain intact under T7 RNA polymerase reaction conditions and that T7 RNA polymerase, though capable of transcribing through crossovers, is increasingly impeded as the number of crossovers increases.

T7GHL BP (buried promoter) was designed to test whether impeding access to the promoter by positioning it in an internal region of a DNA nanoparticle can block transcription. In contrast to expectations, this architecture supported RNA production, but at a slightly reduced level in comparison to the other constructs (Fig. 4). As mentioned above, DNA nanoparticles were observed to maintain structural integrity under IVT reaction conditions as assessed by gel electrophoresis. Yet, to fully exclude the possibility of a “loosened” structure permitting access to the promoter to the T7 RNA polymerase, various concentrations of magnesium ions were introduced into the IVT reaction (Supplementary Fig. 3). The production of RNA was not impacted by the increase in magnesium ion concentration, contradicting the hypothesis that a loosened architecture was the primary reason the buried promoter architecture still permitted transcription. The crystal structure of T7 RNA polymerase reveals its overall dimensions to be 75 Å × 75 Å × 65 Å^35^. The most compact DNA nanoparticle architecture used in this study, T7GHL BP, retains at least 21 bases between any two crossovers connecting adjacent helices, corresponding to a distance of 71.4 Å. Thus, it is reasonable to hypothesize that the T7 RNA polymerase single-subunit enzyme is capable of accessing the T7 promoter inside the structure.

### RT-PCR

**Figure 5:**
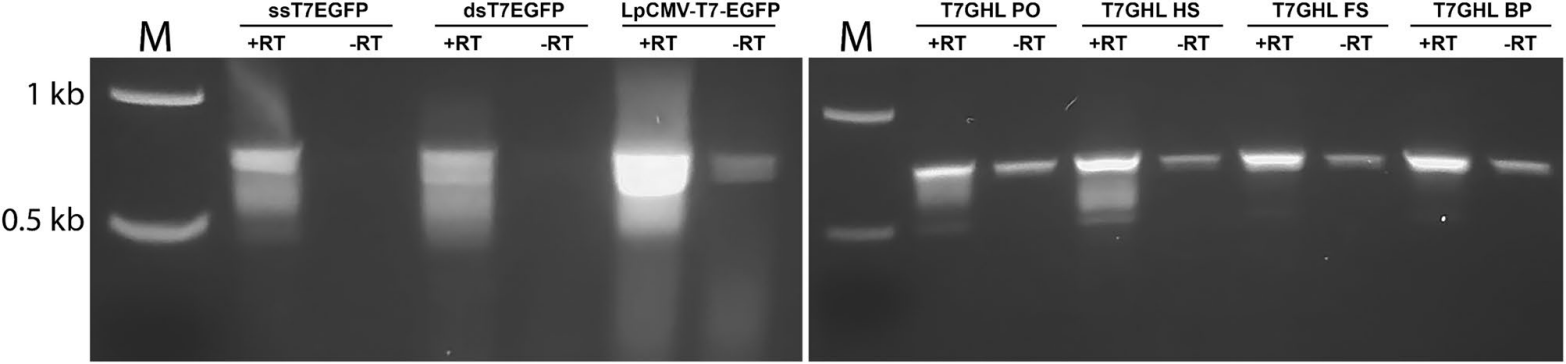
Verification of RNA production by RT-PCR. Representative agarose gel electrophoresis results of RT-PCR. M: molecular marker. + RT: Reverse transcription of RNA products followed by PCR, −RT: PCR of IVT products without reverse transcription of RNA products. The bands around 0.7 kb represent the amplified GFP gene. GFP bands in −RT lanes reflect the amplification of residual DNA that persists after DNase treatment.

To verify that the observed IVT products were indeed GFP RNA transcripts, RT-PCR was performed. Primers were designed to cover the GFP gene. For these experiments, the amount of template used for IVT was titrated to minimize the impact of residual DNA template known to persist even after DNase treatment (NEB personnel communication). To verify that the product of RT-PCR was the result of the amplification of the reverse transcribed DNA, rather than the residual DNA template, identical PCR mixtures were prepared in the absence of reverse transcriptase (negative controls). RT-PCR resulted in DNA bands of approximately 700 bp for all IVT products, which was the expected size for the GFP gene (Fig. 5). Identically sized bands appeared in the control samples, but at a substantially reduced quantity, consistent with them resulting from amplification of residual input DNA. To investigate this further a DNase time course experiment was performed and revealed residual full-length DNA persisted after prolonged DNase treatment (Supplementary Fig. 4). Thus, we conclude that the DNA bands of approximately 700 bp in length are *bona fide* products of reverse transcription of RNA generated in the IVT reactions.

### IVT time course

**Figure 6:**
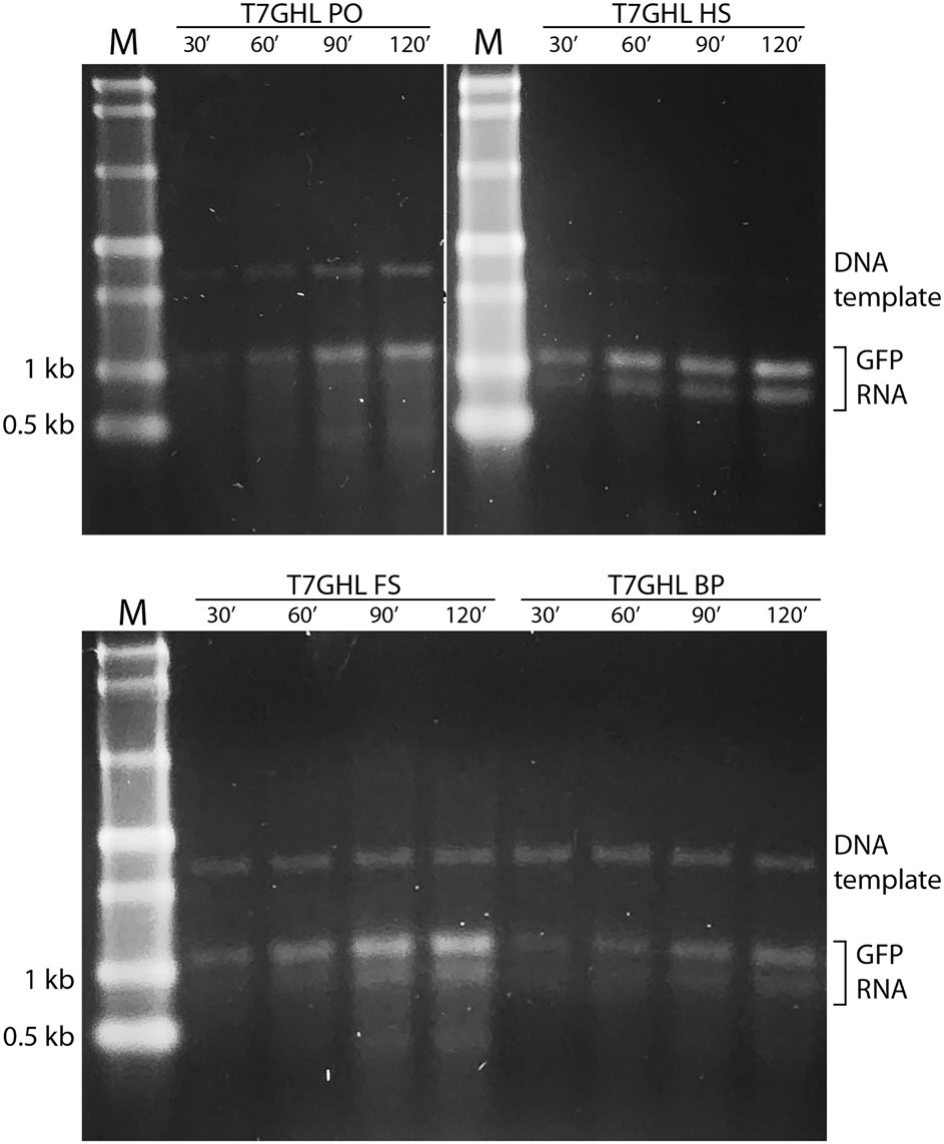
IVT time course with various nanoparticle constructs. Representative non-denaturing agarose gel electrophoresis results of an IVT time course with DNA nanoparticles (T7GHL PO, T7GHL HS, T7GHL FS, and T7GHL BP). M: molecular marker. The bands around 1.1 kb and 0.9 kb represent GFP RNA. The band above the RNA bands (i.e., approximately 2.5 kb) represents the DNA template. For each DNA nanoparticle, IVT reactions were carried out for 30, 60, 90, and 120 min. All DNA nanoparticles used for the time course produced RNA at the earliest time period (30 min), and production of RNA persisted and increased to the 120 min time point.

To assess the persistence/longevity of DNA nanoparticles in IVT reactions, a time course experiment was performed on the various DNA nanoparticle constructs. IVT products were extracted at 30, 60, 90, and 120 min of reaction time. An increase in RNA production was observed with all four promoter-bearing constructs, including the buried promoter, with no obvious variation in transcription kinetics. Thus, it appears that DNA nanoparticles remain transcription competent for at least 120 min in a controlled in vitro environment (Fig. 6).

## Discussion

The self-assembly nanoengineering code embedded in nucleic acids has resulted in the production of DNA nanoparticles with myriad attributes^1,7,21^ and, in some instances, their implementation in various applications^1–3,5–7,10–12,14–18^. The applicability of DNA nanoparticles can be further amplified by the integration of expressible genes in their architecture. This latter point is reflected in a recent report in which a gene cassette folded into a DNA origami nanoparticle was utilized as the substrate for insertion at a genetic locus by a CRISPR/Cas9 editing system in cell culture. In that work, the inserted element was transcribed subsequently to linearization and insertion into the target chromosomal locus^19^. Furthermore, it was recently shown that DNA origami nanoparticles constructed using both sense and antisense gene-bearing scaffold strands can be expressed in cell culture^20^.

In the present study, we explore the possibility that expression can occur within the context of folded DNA nanoparticles that include a significant number of crossovers stereochemically analogous to Holiday junctions. The ability of a polymerase to navigate past a small number of Holliday junctions in linear duplex (i.e., not folded) DNA has been previously described^36^. In that study, it was revealed that Holliday junctions impede, but do not completely block polymerase progression. Here we show that even under conditions of extreme folding, high crossover density, and limited accessibility to the promoter, T7 RNA polymerase can initiate and complete the transcription of a full-length gene cassette. Kretzmann and colleagues have proposed that the unfolding of DNA nanoparticles is a prerequisite for gene expression in cell culture^20^. The study presented here suggests that, at least in vitro, DNA nanoparticles retain their structure prior to transcription and that the unfolding of the DNA nanoparticles occurs during the transcription event. This variation in the proposed mechanism is supported by the AGE results presented in Supplementary materials and the observed trend in differences in RNA production as a function of origami “compactness” (i.e., number, and local density of crossovers). Further delineation of the details involved in gene expression from sculpted DNA nanoparticles may permit regulation of the timing and level of gene expression via fine-tuning of their architectural features.

Finally, it is noteworthy that the chemical malleability of DNA nanoparticles allows them to be readily decorated with a variety of molecular and chemical species, offering mechanisms for enhanced biocompatibility and cellular targeting. For example, electrostatic coating of lipid nanoparticles can significantly enhance their in vivo stability^5,11,37^, various cell-penetrating peptides, as well as nuclear localization signals, can be employed through either conjugation or intercalation for targeted delivery to the nucleus for efficient subsequent gene expression^14,38–41^, and antigen-specific aptamers can be utilized for specific cell targeting^10^. Thus, self-assembling, gene-bearing DNA nanoparticles may constitute the centerpiece of a broadly applicable platform for targeted in vivo delivery of therapeutic proteins^42–44^, protein vaccines^42–45^, and gene editing systems^46–50^.

## Conclusion

In summary, we have demonstrated that gene-bearing, self-assembling DNA nanoparticles formed by the method of DNA origami and containing a large number of crossover domains can be readily transcribed in vitro by T7 RNA polymerase. Crossover density (compactness) and architecturally controlled accessibility to the T7 promoter element can modulate transcription production. In conjunction with previous studies, these results further illustrate the potential of sculpted, gene-bearing, self-assembling DNA nanoparticles to offer significant value in biomedicine and related areas.

### Supplementary Information

Supplementary Information.

## Data Availability

The datasets used and/or analyzed during the current study available from the corresponding author upon request.

## Abbreviations

GFP: Green Fluorescent Protein
IDT: Integrated DNA Technologies
NEB: New England Biolabs
dsDNA: Double-stranded DNA
ssDNA: Single-stranded DNA
dsT7EGFP: Double-stranded GFP gene
ssT7EGFP: Single-stranded GFP gene
IVT: in vitro Transcription
