# Supplementary material for: In vitro transcription of self-assembling DNA nanoparticles
Source: Sci Rep. 2023 Aug 10;13:12961. doi: 10.1038/s41598-023-39777-0 (PMC10415316; doi:10.1038/s41598-023-39777-0)
Supplement: Supplementary file 1 — Supplementary Information. [file 41598_2023_39777_MOESM1_ESM.docx]

**Supporting Information**

The following file is available free of charge.

caDNAno designs of various DNA nanoparticles, their 3D visualizations, and sequences of staples used for the synthesis of each DNA nanoparticle. Supplementary methods and results include verification of structural integrity of DNA nanoparticles under IVT reaction conditions, the effect of additional magnesium ions on RNA production by IVT reactions with DNA nanoparticles, and analysis of residual DNA-based background signal in RT-PCR via extended DNase treatment.

**Materials and Methods**

**In vitro transcription (IVT) on DNA nanoparticles with increasing magnesium (Mg^2+^) concentration.** All IVT reactions were carried out in 20 µL total volume using HiScribe^®^ T7 Quick High Yield RNA Synthesis Kit (NEB). Each reaction component contains 10 µL NTP buffer mix (10 mM each NTP; NEB), 10 ng DNA template (dsT7EGFP and T7GHL BP), 2 µL T7 RNA polymerase mix, 1x TAEM (1x TAE buffer containing 12.5 mM Mg(OAc)­_2_ to give final additional Mg^2+^ concentration of 0, 1.6, 3.2, and 5 mM, and nuclease-free water to volume. For evaluation of structural integrities of DNA nanoparticles in IVT reaction conditions, each reaction was prepared with 10 µL NTP buffer mix (10 mM each NTP; NEB), 10 ng DNA template (T7GHL FS), 1x TAEM to give a final additional Mg^2+^ concentration of 0.9, 1.9, 3.8, and 5 mM, and nuclease-free water to volume. Each reaction was carried out at 37ºC for 2 h. IVT products were purified using Monarch^®^ RNA Cleanup Kit (NEB), electrophoresed, and visualized as described in the manuscript.

**RT-PCR with increasing DNase treatment duration.** For the preparation of substrate for RT-PCR, IVT reactions were performed prior to RT-PCR. All IVT reactions were carried out in 20 µL, containing 10 µL NTP buffer mix (10 mM each NTP; NEB), 1 ng DNA template (dsT7EGFP), 2 µL T7 RNA polymerase mix, and nuclease-free water to volume. All IVT reactions were carried out at 37^o^C overnight. DNase mixtures were prepared by mixing 20 µL of each IVT product, 2 µL DNase-1, and nuclease-free water to volume. DNase treatment was carried out at 37ºC for 0, 5, 15, and 30 min respectively. DNase-treated IVT products were then purified using Monarch^®^ RNA Cleanup Kit (NEB) as described in the paper. RT-PCR was performed using OneTaq^®^ One-step RT-PCR Kit (NEB). All RT-PCR mixtures were carried out in 50 µL. Each mixture contained 1x Quick-Load^®^ OneTaq One-step reaction mix (1.6 mM MgCl_2_, 250 nM dNTP mix; NEB), 400 nM sense primer (RT-sense; IDT), 400 nM antisense primer (RT-anti; IDT), 1 µL each purified RNA product, 1x OneTaq^®^ One-step enzyme mix (ProtoScript^®^ II reverse transcriptase, OneTaq^®^ Hot Start DNA polymerase, Murine RNase inhibitor, and stabilizer), and nuclease-free water to volume. In parallel to RT-PCR mixtures, no RT mixtures (negative controls) were prepared in 50 µL, containing the same components as RT-PCR mixtures. On no RT mixtures, instead of 1x One*Taq*^®^ One-step enzyme mix, 0.5 µL One*Taq*^®^ Hot Start DNA polymerase (NEB) was used. Reverse transcription was carried out by treating each RT-PCR mixture at 48ºC for 30 min followed by PCR. Each PCR was performed using the following thermocycling steps: 30 s at 94ºC, 30 s at 60ºC, and 1 min at 68ºC for 15 cycles. Each product was loaded onto 1% agarose gel pre-stained with SYBR-safe DNA dye (Invitrogen). The gel was electrophoresed and visualized as described in the manuscript.

| 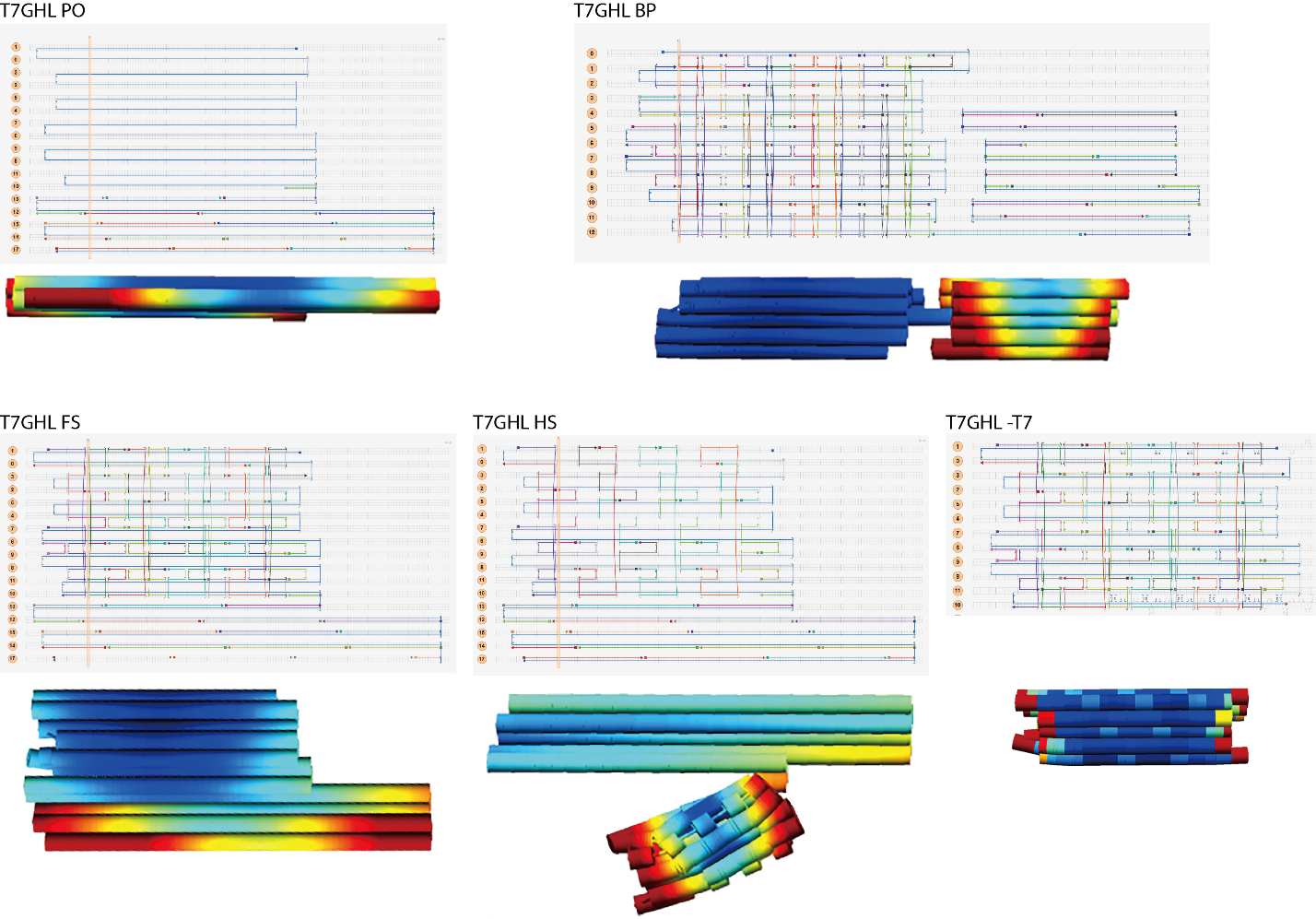 |
| --- |
| **Figure 1.** caDNAno designs of various DNA nanoparticles (GFP gene with T7 promoter with Honeycomb Loop shape structure (T7GHL)). T7GHL PO: DNA nanoparticle with a linear duplex promoter and single-stranded gene; T7GHL HS: DNA nanoparticle with a linear duplex promoter and partially folded gene with half a set of staples; T7GHL FS: DNA nanoparticle with a linear duplex promoter and fully folded gene; T7GHL -T7: fully folded DNA nanoparticle missing T7 promoter; T7GHL BP: DNA nanoparticle with a linear duplex promoter buried inside the fully folded gene. The colors on 3D models indicate relative flexibility within DNA nanoparticles. Red and blue indicate high and low flexibility respectively. |

|  |
| --- |
| **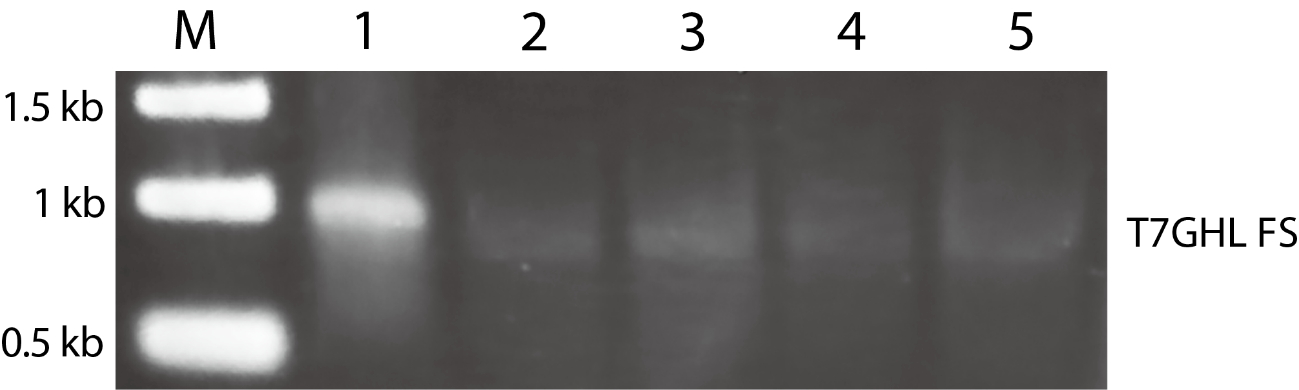** |
| **Figure 2.** Representative agarose gel electrophoresis result verifying the structural integrity of DNA nanoparticle under IVT reaction buffer and temperature with increasing Mg^2+^ concentration. M: molecular marker; 1: T7GHL FS; 2: Treatment of T7GHL FS under IVT reaction buffer and temperature with an additional 0.9 mM Mg^2+^ total; 3: Treatment of T7GHL FS under IVT reaction buffer and temperature T7GHL FS with an additional 1.9 mM Mg^2+^ total; 4: Treatment of T7GHL FS under IVT reaction buffer and temperature T7GHL FS with an additional 3.8 mM Mg^2+^ total; 5: Treatment of T7GHL FS under IVT reaction buffer and temperature T7GHL FS with an additional 5 mM Mg^2+^ total. The bands around 1 kb represent T7GHL FS. The result indicates a minimal shift in DNA nanoparticle migrations suggesting that DNA nanoparticles retain their structural integrities under IVT reaction conditions. |

| 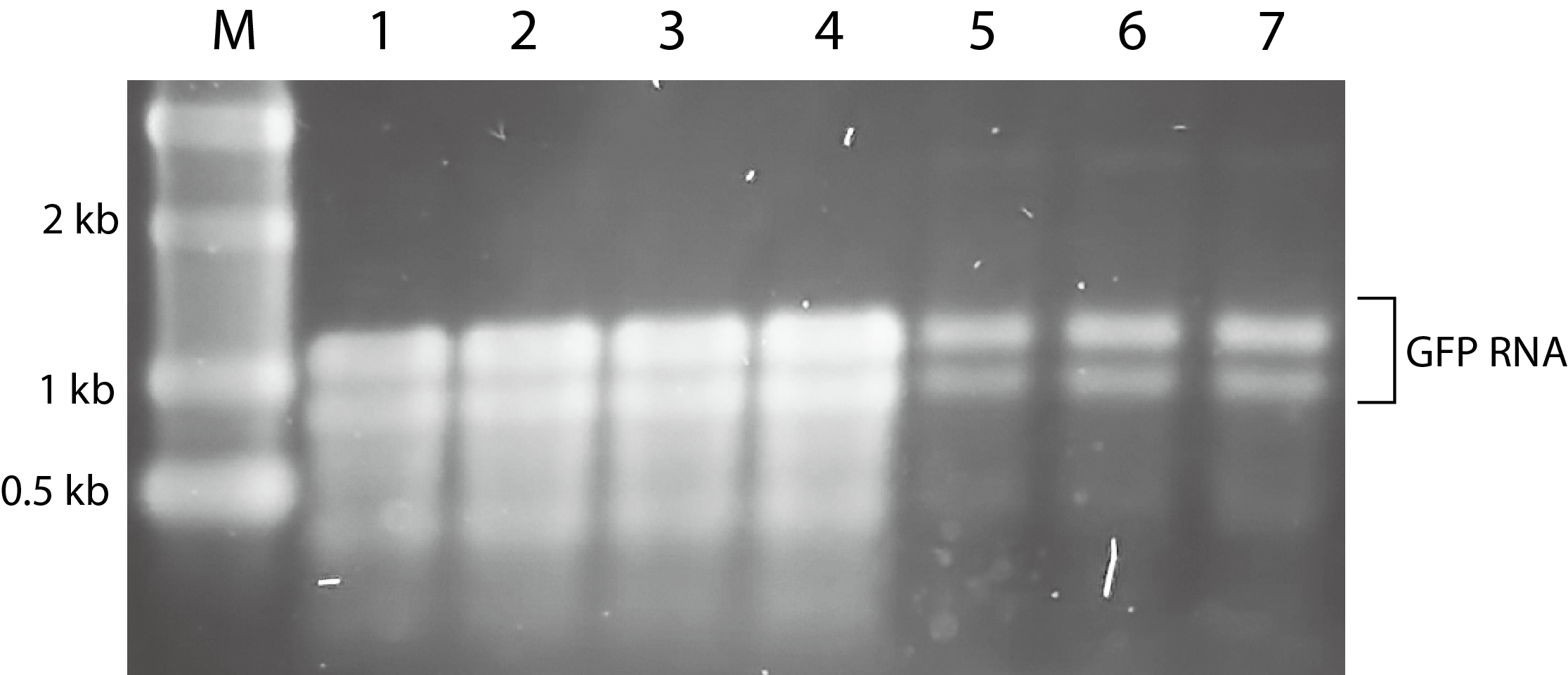 |
| --- |
| **Figure 3.** Representative agarose gel electrophoresis result of products of dsT7EGFP and T7GHL BP IVT with increasing Mg^2+^ concentrations. M: molecular marker; 1: IVT on dsT7EGFP; 2: IVT on dsT7EGFP with an addition of 1.6 mM Mg^2+^; 3: IVT on dsT7EGFP with an addition of 3.2 mM Mg^2+^; 4: IVT on dsT7EGFP with an addition 5 mM Mg^2+^; 5: IVT on T7GHL BP with an addition 1.6 mM Mg^2+^; 6: IVT on dsT7EGFP with an addition 3.2 mM Mg^2+^; 7: IVT on dsT7EGFP with an addition 5 mM Mg^2+^. The bands around 1.1 kb and 0.9 kb represent GFP RNA. Intensities of the bands pre- and post-treatment of additional Mg^2+^ to IVT reactions do not show noticeable differences. Additional magnesium ions were added to IVT reactions to ensure structural integrities of DNA nanoparticles prior to the initiation of the transcription. The result suggests that T7GHL BP remains intact prior to the initiation of the transcription, which further suggests that the transcription of T7GHL BP occurs without pre-disintegration of the nanoparticle structure. |

| 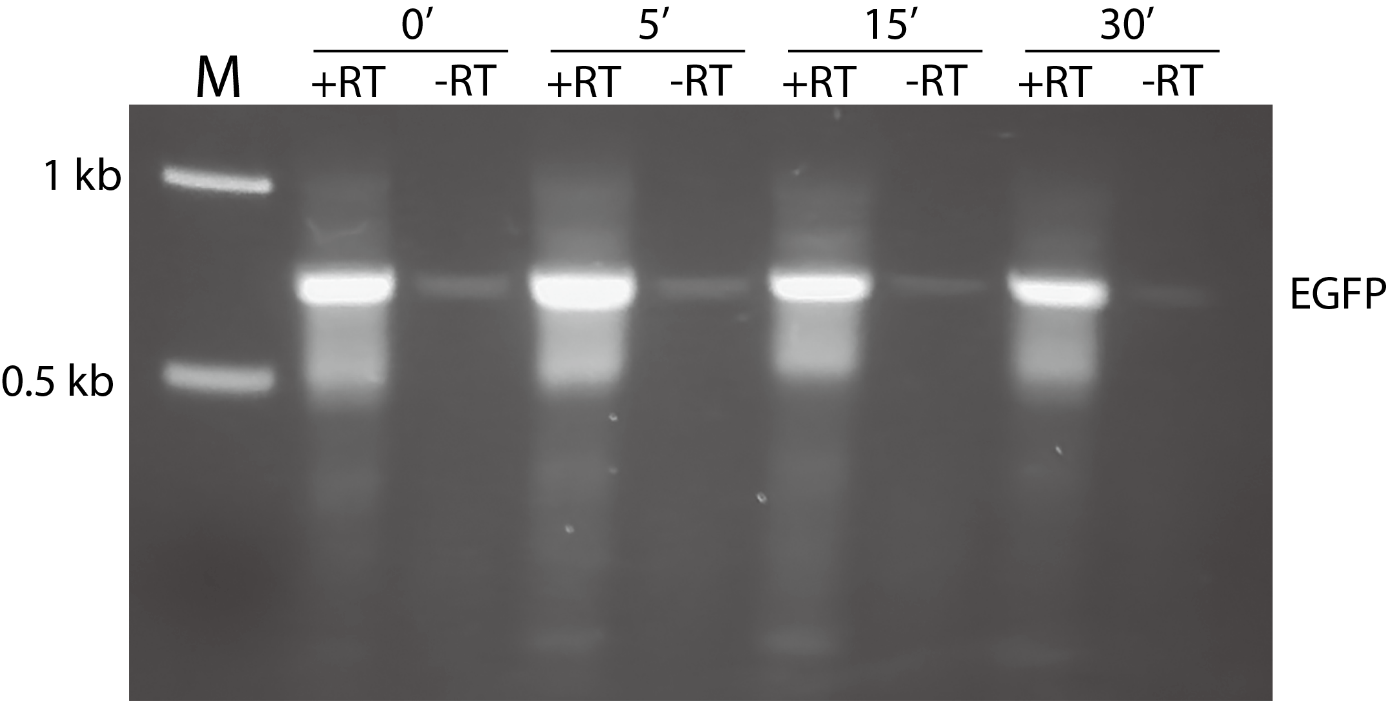 |
| --- |
| **Figure 4.** Representative agarose gel electrophoretic result of RT-PCR products on IVT products with increased DNase treatment time. M: molecular marker; RT: RT-PCR product on IVT with 0 min DNase treatment; PCR product on IVT with 0 min DNase treatment (no RT); RT: RT-PCR product on IVT with 5 min DNase treatment; PCR product on IVT with 5 min DNase treatment (no RT); RT: RT-PCR product on IVT with 15 min DNase treatment; PCR product on IVT with 15 min DNase treatment (no RT); RT: RT-PCR product on IVT with 30 min DNase treatment; PCR product on IVT with 30 min DNase treatment (no RT). The bands around 0.7 kb represent the GFP gene. The intensities of GFP bands on -RT lanes decrease upon increased duration of DNase treatment whereas the intensities of GFP bands on +RT lanes are noticeably bright even upon increased duration of DNase treatment. This supports that background noise on -RT lanes are caused by undegraded full-length DNA template that survived after DNase treatment. |

**Table 1.** Primer sequences for PCR, aPCR, and RT-PCR.

| Primers | Sequences |
| --- | --- |
| T7EGFP sense | 5’ CCA GAT ATA CGC GTT GAC ATT GAT TAT TGA 3’ |
| T7EGFP anti | 5’ TAT ATA CAA TTT CAC ACA GGA AAC AGC TAT GA 3’ |
| 3’ T7EGFP blocker | 5’ CCA GAT ATA CGC GTT GAC ATT GAT TAT TGA /3’phos/ 3’ |
| RT sense | 5’ TGG TGA GCA AGG GCG AGG 3’ |
| RT anti | 5’ TCA CTT GTA CAG CTC GTC CAT GCC 3’ |
| Modifications: /3’phos/: 3’ phosphorylation | |

**Table 2.** T7GHL staple sequences

| Staple names | Sequences |
| --- | --- |
| T7GHL 1 | AAG CTG GAT CCG CCC CTG AGC ACC CAG TGT TCG TG |
| T7GHL 2 | AGT TCC GCG TTA CAT AAC TTA CGG TAA ATG GCC CGC CTG GCT |
| T7GHL 3 | GCT GCC CCC ACC CTC GTG ACC GGG CGA C |
| T7GHL 4 | AGG TGC CGC CAT CTT GCT GGA CCG CCC TAG GTG AA |
| T7GHL 5 | ATG GGT GGA GTA TTT ACG GTA AAC TGC CCA CTT GGC AGT ACA |
| T7GHL 6 | CGG TAG GCG TGT ACG GTG GGA GGT C |
| T7GHL 7 | CGC CGA CTC GAC TTA GTT CGA ACC CTG ATG CAC CA |
| T7GHL 8 | ATC GCT ATT ACC ATG GTG ATG CGG TTT TGG CAG TAC |
| T7GHL 9 | CGA GGA CAC ATC CTA CAA GAC TGC TTC AAA GCT GA |
| T7GHL 10 | AGG ACA GCT GTT TCC TGT GTG GCA AGG GCG AGG AGG TTC ATC CCT ACG G |
| T7GHL 11 | CCA GAT ATA CGC GTT GAC ATT GAT TAT TGA CTA GTT ATT AAT |
| T7GHL 12 | CCC CGA CGG ACG ACG GCA ACT GGG GCA C |
| T7GHL 13 | TGT TCC CAT AGT AAC GCC AAT AGG GAC TTT CCA TTG ACG TCA |
| T7GHL 14 | AAT CAT GCC GGG GTG GTG CCC CTA CGG CGC CGC TA |
| T7GHL 15 | CTT CAA GAG TAC AAT CTT CAA CAC ATG AGA GGG CG |
| T7GHL 16 | GAA GGC TTA TCA TGG C |
| T7GHL 17 | TAT ATA AGC AGA GCT GGT TTA GTG AAC CGT CAG ATC CGC TAG |
| T7GHL 18 | ATG CCA CAT CCT GGC TAG AGC CAA TAG CCT GTC CT |
| T7GHL 19 | CCT CTA GTC GAG CTG GAC GGC CGA GGG CAG CAG CA |
| T7GHL 20 | TTC CTA AGA CTG TGC CGG GAT ACC ACT AAC AAC AT |
| T7GHL 21 | TGT CCG GGA CGT AAC TCG ATA TGC GGT GCC CTG GA |
| T7GHL 22 | AGT AAT CAA TTA CGG GGT CAT TAG TTC ATA GCC CAT ATA TGG |
| T7GHL 23 | AGC CAC AGG CAT CAG AGC AAA GAC CCC ACG ATC ACA TGG TCC GTT GTT T |
| T7GHL 24 | GAC CGC CCA ACG ACC CCC GCC CAA TGA CGT CAA TAA TGA CGT A |
| T7GHL 25 | AGA ACC AGC TGG GGA CGG CCA CA |
| T7GHL 26 | AGA TCC GCG GCC GCT AAT ACG ACT CAC TAT AGG GAG AGC CGC CAC C |
| T7GHL 27 | TCA AGT GTA TCA TAT GCC AAG TAC GCC CCC TAT TGA CGT CAA T |
| T7GHL 28 | GAC GGC AGG CAG CGG CTG CTG CCC GAC ACA CTC TC |
| T7GHL 29 | AGT CTC CAC CCC ATT GAC GTC AAT GGG AGT TTG TTT TGG CAC |
| T7GHL 30 | CGT GCA GCC GCG CCG AGG TGA CAA GGA G |
| T7GHL 31 | ACA AGC AGA AGA ACA CGT CTA ACG TCC ATT CAA GTT TCA GCG |
| T7GHL 32 | GAC GGT AAA TGG CCC GCC TGG CAT TAT GCC CAG TAC ATG ACC |
| T7GHL 33 | ATC AAT GGG CGT GGA TAG CGG TTT GAC TCA CGG GGA TTT CCA |
| T7GHL 34 | TTA TGG GAC TTT CCT ACT TGG CAG TAC ATC TAC GTA TTA GTC |
| T7GHL 35 | CCA TTG ACG CAA ATG GG |
| T7GHL 36 | CCC TGA ACT GTT CAG TCA TAG CAA GGG GAG GAA AT |
| T7GHL 37 | TGC ATC GTT AAA CCA CGA GCT GCC CCG TTG CAG CT |
| T7GHL 38 | CGA CTT CGG AGC GCA CCA TCT CTA CAA C |
| T7GHL 39 | GGC ATG GCG CTG ATC AGC CTC TAA AAT GGA GGA TTG GGA AGA TTG GCG T |
| T7GHL 40 | ACC CTG GCG AGC TGA AGG GCA CAC TAC CCC CCA TCG GCG ACG GTA CAA G |
| T7GHL 41 | GCC CCT CTC CTT GAG GCT CTA TGG CTT CTG AGG |
| T7GHL 42 | CAA AAT CAA CGG GAC TTT CCA AAA TGT CGT AAC AAC TCC GCC |
| T7GHL 43 | TGA CCG GCA TCA CCA TTG AGT CAT TGT CGT GGG GC |
| T7GHL 44 | ACC GCC GCC TTC TAG TTG CCA ACT CCC AAG GCA TGC TGG GGA CCG TCG A |
| T7GHL 45 | CCG GCA AAT GGT GAA AAT TGT GGG TGG GTG AGT AGG TGT CAT |

**Table 3.** T7GHL BP staple sequences

| Staple name | Sequence |
| --- | --- |
| T7GHL BP 1 | GCA GCA CTG TCC GGG ACG TAA CTC GAT ATG CGG TG |
| T7GHL BP 2 | AAG CTG AGT GCA GTG GAC GAC |
| T7GHL BP 3 | CTG TGT GGC AAG GGA TAG GGA AGG GCA TGG CAG CG |
| T7GHL BP 4 | CCA AGT CTC CAC CCC ATT GAC GTC AAT GGG AGT TTG TTT TGG C |
| T7GHL BP 5 | TGC ACC AGT GAC CAC CGC GCC |
| T7GHL BP 6 | GTA ACG CCA ATA GGG ACT TTC CAT TGA CGT CAA TGG GTG GAG TAT T |
| T7GHL BP 7 | GAG GAT TTC CTA ATA CTG TGC CGG GAT CCC ACT AC |
| T7GHL BP 8 | ACA TCT ACG TAT TAG TCA TCG CTA TTA CCA TGG TGA TGC GGT TTT G |
| T7GHL BP 9 | ACC AAA ATC AAC GGG ACT TTC CAA AAT GTC GTA ACA ACT CCG CCC CA |
| T7GHL BP 10 | GGC AAC TAA GGA GGT AAT ACG AGC CTC GAA AAT GA |
| T7GHL BP 11 | CAC CCT CCC GGC AAA TGG TGA AAA TTG TGG GTG GG |
| T7GHL BP 12 | AAG TAC GCC CCC TAT TGA CGT CAA TGA CGG TAA ATG GCC CGC CTG G |
| T7GHL BP 13 | GTG GGG CGC ATC GCT AAA CCC CGA GCT GCC CCG TG |
| T7GHL BP 14 | GCA TGG AGC TGA TCA CTC ACT CGA GGA GGT TCA TC |
| T7GHL BP 15 | GGA AAT TAG GAC AGC TGT TTC |
| T7GHL BP 16 | TTG ACG CAA ATG GGC GGT AGG CGT GTA CGG TGG GAG GTC TAT ATA A |
| T7GHL BP 17 | GAG GGC GCC CGA CCG GAG CGC |
| T7GHL BP 18 | AGT AAT CAA TTA CGG GGT CAT TAG TTC ATA GCC CAT ATA TGG A |
| T7GHL BP 19 | CCT GGA ACT GGG GAC CGT CGA |
| T7GHL BP 20 | CCA GAT ATA CGC GTT GAC ATT GAT TAT TGA CTA GTT ATT AAT |
| T7GHL BP 21 | TGG TCC TTT GTT TGG TGA ACC GGA CGG CCG AGG GC |
| T7GHL BP 22 | CCG CTA CAT GCC ACA TCC TGG CTA GAG CCA ATA GC |
| T7GHL BP 23 | GAA GGC TTA CAA CAT GGT TTA CCC CTC CCC TTG AC |
| T7GHL BP 24 | CCT GGT GAA CAC TAC CAG |
| T7GHL BP 25 | CCG CCG CCT TCT AGA GAT CCG GGT GCC CCT ACG GC |
| T7GHL BP 26 | GTT CCG CGT TAC ATA ACT TAC GGT AAA TGG CCC GCC TGG CTG ACC GC |
| T7GHL BP 27 | CTG AGC ACT TCA AGC ATC CTG TCT TCA AGC TTC AG |
| T7GHL BP 28 | ACA ACA TCC GAC AAA CTC TCG |
| T7GHL BP 29 | CCT CTA GTC GAG CTG TCA GAT AGC TGG AGG CAT CA |
| T7GHL BP 30 | AAT CAT GCC GGG GTC GGC CGC ACG GCA AAT CCG CC |
| T7GHL BP 31 | CTA CGG CCC CTG AAC TGT TCA GTC ATA GCA AGG GG |
| T7GHL BP 32 | TTC AGC GGA CTT CTC ATG CCC |
| T7GHL BP 33 | GGC TCT ATG GCT TCA GAA CCA |
| T7GHL BP 34 | GCA GTA CAT CAA TGG GCG TGG ATA GCG GTT TGA CTC ACG GGG ATT T |
| T7GHL BP 35 | GAA CAC CCC CAT CGC GCC GAC CCG CAT CAG TTC GAC CTG GCC |
| T7GHL BP 36 | CCA ACG ACC CCC GCC CAA TGA CGT CAA TAA TGA CGT ATG TTC CCA TA |
| T7GHL BP 37 | TGT CCT TGG GAA GAT TGG CGT |
| T7GHL BP 38 | GAC CGG TCA TCA TCA CCA TCA CCA |
| T7GHL BP 39 | CTG CTG CCG AGG ACC GAC TTC ACA AGA CCC CTG AC |
| T7GHL BP 40 | CAT TAT GCC CAG TAC ATG ACC TTA TGG GAC TTT CCT ACT TGG CAG T |
| T7GHL BP 41 | AGC AAA GGA AGA ACG TAC AAC ACG TCC AAC ATG AA |
| T7GHL BP 42 | ACC ATC TGG GCA CAC CGC TAG TTG CCA GCT CCC AC |
| T7GHL BP 43 | GAG GTG AGA GCT GAG AGC CGC TTG AGT TAT TGT CTG AGT AGG TTC TGG G |
| T7GHL BP 44 | TAC GGT AAA CTG CCC ACT TGG CAG TAC ATC AAG TGT ATC ATA TGC C |
| T7GHL BP 45 | GCT GGG GAC GGC CAG CAG AGC GCC ACA AAT GGC CG |
| T7GHL BP 46 | AGG CAT GGG TGC CAC CAT CTG GCT GGA GCG CCC TG |
| T7GHL BP 47 | TGC AGC TGC GAC GGT ACA AGT |
| T7GHL BP 48 | ACA AGC AAC CCC AAG ATC ACA |
| T7GHL BP 49 | AGG TGA ACC CAG TCT TCG TGA |

**Table 4.** Staple list for DNA nanoparticle construction

| DNA nanoparticle | Staple list |
| --- | --- |
| T7GHL PO | T7GHL 2,5,6,8,11,13,17,22,24,26,27,29,32-35,42 |
| T7GHL HS | T7GHL 1-3,5,6,8,10-14,17,19,22-35,38-44 |
| T7GHL FS | T7GHL 1-45 |
| T7GHL BP | T7GHL BP 1-49 |
| T7GHL-T7 | T7GHL 1-45 |
